# Opportunistic Capacity-Based Resource Allocation for Chunk-Based Multi-Carrier Cognitive Radio Sensor Networks

**DOI:** 10.3390/s17010175

**Published:** 2017-01-18

**Authors:** Jie Huang, Xiaoping Zeng, Xin Jian, Xiaoheng Tan, Qi Zhang

**Affiliations:** College of Communication Engineering, Chongqing University, Chongqing 400044, China; 20111202042@cqu.edu.cn (J.H.); zxp@cqu.edu.cn (X.Z.); txh@cqu.edu.cn (X.T.); 20104710@cqu.edu.cn (Q.Z.)

**Keywords:** cognitive radio sensor networks, spectrum holes, resource allocation, opportunity capacity

## Abstract

The spectrum allocation for cognitive radio sensor networks (CRSNs) has received considerable research attention under the assumption that the spectrum environment is static. However, in practice, the spectrum environment varies over time due to primary user/secondary user (PU/SU) activity and mobility, resulting in time-varied spectrum resources. This paper studies resource allocation for chunk-based multi-carrier CRSNs with time-varied spectrum resources. We present a novel opportunistic capacity model through a continuous time semi-Markov chain (CTSMC) to describe the time-varied spectrum resources of chunks and, based on this, a joint power and chunk allocation model by considering the opportunistically available capacity of chunks is proposed. To reduce the computational complexity, we split this model into two sub-problems and solve them via the Lagrangian dual method. Simulation results illustrate that the proposed opportunistic capacity-based resource allocation algorithm can achieve better performance compared with traditional algorithms when the spectrum environment is time-varied.

## 1. Introduction

A wireless sensor network (WSN) is a wireless network that consists of a large number of sensor nodes that can be applied to environmental monitoring, invasion detection, disaster aid and various other fields [1,2]. Current WSNs operate in the industrial, scientific and medical (ISM) band, which is shared by many other successful communication technologies. With emerging broadband applications of wireless communications, the radio spectrum resources for WSNs in the ISM band are currently suffering from serious shortages, which degrades the performance of the WSNs [3]. This frequency band problem is exacerbated because radio spectrum is becoming an increasingly important and scarce resource in wireless communication systems. However, because of the current fixed spectrum assignment policy, the spectrum utilization efficiency in the licensed spectrum is very low, which generates many non-continuous vacant frequency bands, referred to as spectrum holes [4,5]. To overcome the spectrum resource limitation experienced by current WSNs and improve the spectrum utilization, cognitive radio (CR), which allows secondary users (SUs) to opportunistically utilize the frequency spectrum originally assigned to licensed primary users (PUs), has been used in WSNs as a promising approach to alleviate spectrum scarcity [6,7,8]. A WSN in which the sensor nodes are equipped with cognitive radios is called a cognitive radio sensor network (CRSN) [9,10,11]. CRSNs are a candidate area in which cognitive techniques can be used to provide opportunistic spectrum access.

In CRSNs, resource allocation is an important and challenging task that aims to assign discrete spectrum resources to achieve efficient frequency utilization and maximize the capacity. In the existing literature, considerable achievements have been made for resource allocation in CRSNs by applying methods from game theory [12,13], graph theory [14,15] and linear programming [16,17,18]. These studies laid the foundation for the researches of resource allocation, but few of them consider the dynamic available capacity of spectrum resources. Recently, the dynamic available capacity of spectrum resources have received considerable attention from academia, which has focused mainly on the time-varied available resources of the sub-carrier, also known as opportunistic capacity [19]. In [20], the authors modeled the PU activity in a sub-carrier as a semi-Markov ON/OFF process using varying distributions of holding times (ON and OFF states) and studied the impact of activity model parameters on spectrum efficiency. Subsequently, [21] studied the PU and SU activity and modeled the busy/idle times in a sub-carrier using two-state Markov chains. In [22], the authors modeled the PUs as independent M/G/1 queues with Poisson packet arrival rates and investigated the channel access problem by deriving the probabilities of collisions vs. successful transmissions for the PUs and SUs. In summary, most existing works focus more on the opportunistic capacity of a single sub-carrier. However, in practice, a set of contiguous sub-carriers is often grouped into one chunk and then allocated in a chunk-by-chunk manner to users for simplification, because the number of sub-carriers is very large in a multi-carrier communication system [23,24,25,26,27]. Similar to the time-varied available resources of a single sub-carrier, the available resources of chunks are also time-varied in chunk-based multi-carrier CRSNs. Therefore, exploring a resource allocation algorithm by considering the opportunistic capacity of chunks is necessary for chunk-based multi-carrier CRSNs. Achieving such resource allocation will reduce spectrum collisions and greatly improve spectrum efficiency. To the best of our knowledge, resource allocation in chunk-based multi-carrier CRSNs by considering opportunistic capacity of chunks has not been studied in previous works.

This paper studies the resource allocation for chunk-based multi-carrier CRSNs where the available spectrum resources are time-varied due to PU/SU activity and mobility. We present a novel opportunistic capacity model through continuous time semi-Markov chain (CTSMC) to describe the time-varied spectrum resources of chunks. This model is different from existing studies that focus primarily on the opportunistic capacity of a single sub-carrier. Using our method, a joint power and chunk allocation model based on the opportunistic capacity of chunks is established for chunk-based multi-carrier CRSNs with time-varied spectrum resources. To reduce the computational complexity, we split this model into two sub-problems and solve them using the Lagrangian dual method. Simulation results illustrate that the proposed opportunistic capacity-based resource allocation can achieve better performance compared with traditional algorithms when the spectrum environment is time-varied.

The remainder of this paper is organized as follows. The system model is described in Section 2. Section 3 presents the opportunistic capacity model for chunks. In Section 4, the resource allocation algorithm based on the opportunistic capacity of chunks is proposed. Numerical results are provided in Section 5 to demonstrate the advantages of the proposed scheme. We conclude this paper in Section 6.

## 2. System Model

**Figure 1:**
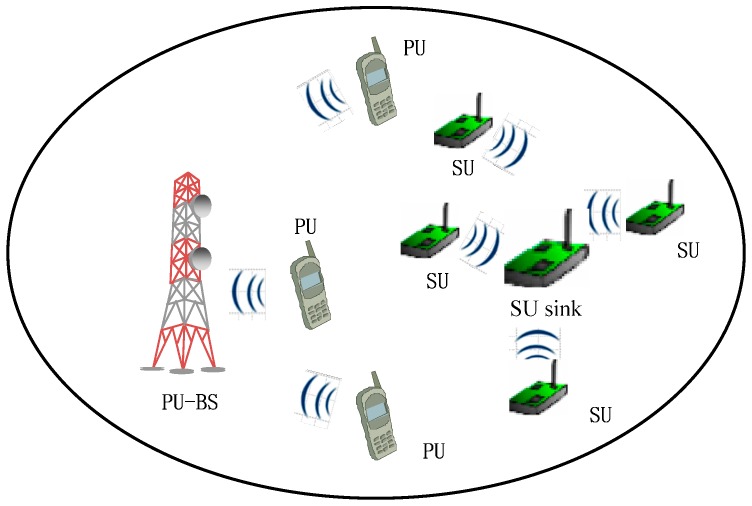
System model of cognitive radio sensor networks (CRSNs).

In this work, we consider chunk-based multi-carrier CRSNs, where a set of sensor nodes are communicating with a centralized secondary user sink (SU sink) that is either the cluster head or a secondary base station. Because the sensor nodes communicate using the frequency band licensed to the PU, we call these secondary sensor nodes SUs. The CRSNs we consider are shown in Figure 1, which includes PUs, SUs, PUs’ base stations and an SU sink. In this system, the SUs adopt a centralized chunk-based resource allocation scheme and opportunistically utilize the holes in the spectrum through spectrum sensing [28]. In the spectrum sensing phase, SUs periodically detect information concerning the local frequency spectrum and send it to the SU sink. Then, the SU sink summarizes all the detection information sent by the SUs to find the spectrum holes and divide them into chunks, the minimum units for allocation. It allocates spectrum to SUs in a chunk-by-chunk manner by considering the different needs of SUs. We assume that the spectrum environment is time-varied due to PU/SU activity and mobility, which will lead to frequent busy/idle changes in each sub-carrier and a time-varied number of idle sub-carriers in the chunk. These time-varied spectrum resources make resource allocation quite difficult. To address this issue, we formulate an opportunistic capacity model for chunks that describes the time-varied spectrum resources. Then, based on this, we propose a joint power and chunk allocation algorithm for chunk-based multi-carrier CRSNs.

## 3. Opportunistic Capacity Model for Chunks

In this section, the time-varied spectrum resources of chunks have been formulated mathematically. Each chunk is assumed to have n sub-carriers that support a certain frequency range. Due to the time-varied spectrum environment, each sub-carrier can be in one of two states, busy or idle, and the number of idle sub-carriers in each chunk changes over time. We consider the number of available idle sub-carriers in a chunk as the chunk state; then, based on the properties of the state transition model, we can derive the opportunistic capacity for the chunk. We assume the number of idle sub-carriers in a chunk at a certain time t is $X(t)=S_{i}=i,\;(0\leq i\leq n)$, which also represents the instantaneous available capacity in the chunk at time t. Because i sub-carriers are not being used by the PUs in state $S_{i}$, $S_{i}$ is decremented to $S_{i-1}$ if a PU transmission arrives at any of these sub-carriers. In contrast, $S_{i}$ can be incremented to $S_{i+1}$ if one of the n−i PUs in service completes its transmission and releases the sub-carrier. Based on this, a chunk can transition between states only during PU arrivals or departures in the chunk. This state transition model can be described by the embedded Markov method, whose expectation of the number of idle sub-carriers in a chunk is that chunk’s opportunistic capacity.

**Figure 2:**
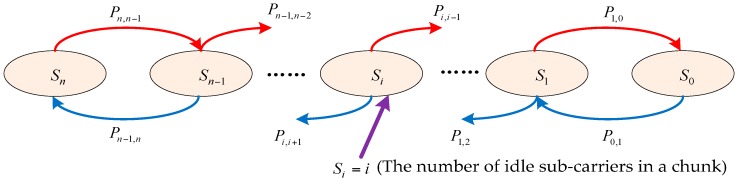
The state transition model for the number of idle sub-carriers in a chunk.

The state transition model for the number of idle sub-carriers in a chunk is depicted in Figure 2. The state space $S=\{S_{0},S_{1}\ldots ,S_{n}\}$ consists of the state $S_{i}=i$ where 0≤i≤n, which denotes that the number of idle sub-carriers in the chunk is i. $P_{i,i-1}$ denotes the transition probability that a PU arrival in any of *i* idle sub-carriers in state $S_{i}$, while $P_{i,i+1}$ represents the transition probability that one of the n−i PUs in a busy sub-carrier completes its service and releases the sub-carrier. We can assume that the holding time of the PUs in each sub-carrier follows an exponential distribution with expectation $\frac{1}{\lambda }$ and that the idle time in each sub-carrier follows a Pareto distribution $Pa(a,t_{m})$ because [29] analyzed real-world empirical data and concluded that a Pareto distribution function can easily describe the idle time for all the considered bands at long timescales. The memory property of the Pareto distribution transmits the state transition model into a CTSMC model, which makes it quite complicated, because the remaining idle time of the idle period at a given idle time, t, is related to the past idle time for each sub-carrier. These kinds of CTSMC models do not have a general solution at present. Thus, to reduce the complexity, we tackle this issue by approximating the probability density function (PDF) of the remaining idle time of the idle period in the sub-carrier. According to the method in [30], the cumulative distribution function (CDF) of the remaining idle time t can be approximated as follows:

(1) $$G_{e}(t)=\frac{\int_{0}^{t}\left[1-G(u)\right]du}{E[G(t)]},$$

where G(t) indicates the CDF of the idle period in the sub-carrier, and E[G(t)] denotes the expectation of the idle period. Substituting the Pareto distribution $Pa(a,t_{m})$ into Equation (1) yields

(2) $$G_{e}(t)=\left\{ \begin{array}{ll} \frac{a-1}{a}+\frac{1}{a}(1-\frac{{t_{m}}^{a-1}}{t^{a-1}}) & t>t_{m}\\ \frac{(a-1)t}{at_{m}} & t<t_{m} \end{array}\right..$$

Now $G_{e}(t)$ can denote the CDF of the remaining idle time t, and the PDF of the remaining idle time can be given as follows:

(3) $$g_{e}(t)=\left\{ \begin{array}{ll} \frac{(a-1){t_{m}}^{a-1}}{at^{a}} & t>t_{m}\\ \frac{a-1}{at_{m}} & t<t_{m} \end{array}\right..$$

Therefore, the expectation of the remaining idle time can be achieved by calculating

(4) $$E[G_{e}(t)]=\int_{0}^{+\infty }tg(t)dt=\int_{0}^{t_{m}}\frac{a-1}{at_{m}}tdt+\int_{t_{m}}^{+\infty }\frac{(a-1){t_{m}}^{a-1}}{at^{a-1}}dt=\frac{(a-1)t_{m}}{2(a-2)}.$$

Then, the transition rate of the idle sub-carrier, which is the reciprocal of the remaining idle time, can be approximated by

(5) $$\nu =\frac{2(a-2)}{(a-1)t_{m}}.$$

Based on the memorylessness property of exponential distribution, the transition rate of the busy sub-carrier is λ. Therefore, $P_{i,i+1}$ and $P_{i,i-1}$ in state $S_{i}$ can be expressed as follows:

(6) $$P_{i,i+1}=\frac{\lambda t_{m}(a-1)(n-i)}{\lambda t_{m}(a-1)(n-i)+2i(a-2)},$$

and

(7) $$P_{i,i-1}=\frac{2i(a-2)}{\lambda t_{m}(a-1)(n-i)+2i(a-2)}.$$

Then, the state transition matrix P can be given:

(8) $$P=\left[\begin{array}{cccccc} 0 & 1 & 0 & & & \\ P_{1,0} & 0 & P_{1,2} & 0 & & \\ & \ddots & \ddots & \ddots & & \\ & & P_{i,i-1} & 0 & P_{i,i+1} & \\ & & \ddots & \ddots & \ddots & \\ & & & & 1 & 0 \end{array}\right].$$

Therefore, the stationary probability $\pi =(\pi_{1},\pi_{2},\ldots ,\pi_{n})$, which represents the proportion of transitions that take the process into state $S_{i},(0\leq i\leq n)$ can be obtained by solving the following equations:

(9) πP=π,

and

(10) $$\sum_{i=0}^{n}\pi_{i}=1.$$

Subsequently, according to [31], the limiting probability $P_{i}$, which represents the proportion of time that the process is in state $S_{i}$, can be calculated by

(11) $$P_{i}=\frac{\pi_{i}\mu_{i}}{\sum_{j=1}^{n}\pi_{j}\mu_{j}},$$

where $\mu_{i}$ is the expectation of residence time in state $S_{i}$ during each visit. Because the end of state $S_{i}$ can occur only at times of PU arrival and PU departure in the chunk, $\mu_{i}$ can be written as

(12) $$\mu_{i}=p_{i,i+1}E[h_{i,i+1}(t)]+p_{i,i-1}E[h_{i,i-1}(t)],$$

where $h_{i,i+1}(t)$ and $h_{i,i-1}(t)$ are the PDFs of residence times in state $S_{i}$ before transition to the states $S_{i+1}$ and $S_{i-1}$, respectively, and $E[h_{i,i+1}(t)]$ and $E[h_{i,i-1}(t)]$ represent the expectations of residence time in state $S_{i}$ before transition to the states $S_{i+1}$ and $S_{i-1}$, respectively. The residence time in state $S_{i}$ before transition to state $S_{i-1}$ can be expressed by the minimum remaining idle time of i idle sub-carriers, because transition can occur only at the time that a PU arrives at one of the idle sub-carriers. Assume that the remaining idle time of each idle sub-carrier be a random variable. Then $h_{i,i-1}(t)$ can be given as the PDF of the minimum order statistic of i random variables. Similarly, $h_{i,i+1}(t)$ can be given as the PDF of the minimum order statistic of n−i random variables, representing the remaining busy time of busy sub-carriers. According to the minimum order statistic [32], $h_{i,i+1}(t)$ and $h_{i,i-1}(t)$ can be written as

(13) $$h_{i,i-1}(t)=\left\{ \begin{array}{ll} \frac{i(a-1){t_{m}}^{i(a-1)}}{a^{i}t^{i(a-1)+1}} & t>t_{m}\\ iq{\left[1-qt\right]}^{i-1} & t\leq t_{m} \end{array}\right.,$$

and

(14) $$h_{i,i+1}(t)=(n-i)\lambda e^{-\lambda (n-i)t},$$

respectively, where $q=\frac{a-1}{at_{m}}$. Then, $E[h_{i,i+1}(t)]$ and $E[h_{i,i-1}(t)]$ can be obtained as follows:

(15) $$\begin{array}{cc} E[h_{i,i-1}(t)] & =\int_{0}^{t_{m}}iqt{\left[1-qt\right]}^{i-1}dt+\int_{t_{m}}^{+\infty }\frac{i(a-1){t_{m}}^{i(a-1)}}{a^{i}t^{i(a-1)}}dt\\ & =iq\int_{0}^{t_{m}}t\sum_{k=0}^{i}\frac{i!{\left(-q\right)}^{k}t^{k}}{(i-k)!k!}dt+\frac{i(a-1){t_{m}}^{i(a-1)}}{a^{i}}\int_{t_{m}}^{+\infty }\frac{1}{t^{i(a-1)}}dt\\ & =iq\sum_{k=0}^{i}\frac{i!{\left(-q\right)}^{k}{t_{m}}^{k+2}}{\left(i-k)!k!(k+2\right)}+\frac{i(a-1)t_{m}}{a^{i}(ia-i-1)} \end{array},$$

(16) $$E[h_{i,i+1}(t)]=\int_{0}^{+\infty }(n-i)\lambda e^{-\lambda (n-i)t}dt=\frac{1}{\lambda (n-i)}.$$

Substituting Equations (6), (7), (15) and (16) into Equation (12) yields

(17) $$\mu_{i}=\frac{\left(a-1)\{at_{m}+2i^{2}(a-2)[\sum_{k=0}^{i}\frac{i!{\left(-q\right)}^{k}{t_{m}}^{k+1}}{\left(i-k)!k!(k+2\right)}+\frac{i(a-1)}{a^{i}(ia-i-1)}]\right\}}{a[\lambda t_{m}(a-1)(n-i)+2i(a-2)]}.$$

By bringing $\mu_{i}$ and $\pi_{i}$ into Equation (11), the result is

(18) $$P_{i}=\frac{\frac{\pi_{i}(a-1)\{at_{m}+2i^{2}(a-2)[\sum_{k=0}^{i}\frac{k!{\left(-q\right)}^{k}{t_{m}}^{k+1}}{\left(i-k)!k!(k+2\right)}+\frac{i(a-1)}{a^{i}(ia-i-1)}]\}}{a[\lambda t_{m}(a-1)(n-i)+2i(a-2)]}}{\sum_{j=1}^{n}\frac{\pi_{j}(a-1)\{at_{m}+2j^{2}(a-2)[\sum_{k=0}^{j}\frac{j!{\left(-q\right)}^{k}{t_{m}}^{k+1}}{\left(j-k)!k!(k+2\right)}+\frac{j(a-1)}{a^{j}(ja-j-1)}]\}}{a[\lambda t_{m}(a-1)(n-j)+2j(a-2)]}}.$$

Based on this, the opportunistic capacity that denotes the expectation of the number of idle sub-carriers in a chunk can be calculated as follows:

(19) $$C=\sum_{i=0}^{n}iP_{i}=\sum_{i=0}^{n}\frac{\frac{i\pi_{i}(a-1)\{at_{m}+2i^{2}(a-2)[\sum_{k=0}^{i}\frac{k!{\left(-q\right)}^{k}{t_{m}}^{k+1}}{\left(i-k)!k!(k+2\right)}+\frac{i(a-1)}{a^{i}(ia-i-1)}]\}}{a[\lambda t_{m}(a-1)(n-i)+2i(a-2)]}}{\sum_{j=1}^{n}\frac{\pi_{j}(a-1)\{at_{m}+2j^{2}(a-2)[\sum_{k=0}^{j}\frac{j!{\left(-q\right)}^{k}{t_{m}}^{k+1}}{\left(j-k)!k!(k+2\right)}+\frac{j(a-1)}{a^{j}(ja-j-1)}]\}}{a[\lambda t_{m}(a-1)(n-j)+2j(a-2)]}}.$$

## 4. Opportunistic Capacity-Based Resource Allocation for Chunk-Based Multi-Carrier CRSNs

### 4.1. Opportunistic Capacity-Based Resource Allocation Model

We assume that the CRSNs consist of m SUs and k idle chunks with total transmit power constraint $P_{T}$. Among these, each chunk contains n sub-carriers, and each SU i (i=1,2,3,⋅⋅⋅,m) can utilize multiple chunks to ensure that its data rate requirements indicated by $Q=\{q_{1},\ldots ,q_{m}\}$ can be met. To consider the statistical property of available capacity in the chunk at long timescales, we introduce $C=\{c_{1},\ldots ,c_{k}\}$ ($c_{j}\leq n$, j=1,2,3,⋅⋅⋅,k) as the opportunistic capacity of chunks and make the total opportunistic capacity assigned to each SU satisfy that SU’s data rate requirement. This opportunistic capacity-based resource allocation can reduce spectrum collisions and greatly improve spectrum efficiency under a time-varied spectrum environment (e.g., allocating chunks with small opportunistic capacity to SUs that have small data rates demands to make full use of spectrum fragments). The joint power and chunk allocation model can be transformed into a combinatorial optimization problem to achieve the maximum transmission rate:

(20) $$\max\;\sum_{i=1}^{m}\sum_{j=1}^{k}c_{j}r_{i,j}x_{i,j},$$

(21) $$s.t.\;\sum_{i=1}^{m}\sum_{j=1}^{k}x_{i,j}p_{i,j}\leq P_{T},$$

(22) $$\sum_{j=1}^{k}c_{j}r_{i,j}x_{i,j}\geq q_{i},$$

and

(23) $$\sum_{i=1}^{m}x_{i,j}\leq 1.$$

Let $x_{i,j}$ indicate whether chunk j is allocated to SU i. If chunk j is allocated to SU i, $x_{i,j}=1$; otherwise, $x_{i,j}=0$. Then, $p_{i,j}$ denotes the transmit power allocated to SU i when SU i transmits on chunk j, and $r_{i,j}$ represents the data rate per sub-carrier in chunk j for SU i because the channel fading within the same chunk is assumed to be the same. Constraint (21) ensures that the total transmit power satisfies the power constraint. Constraint (22) ensures that the total opportunistic capacity assigned to each SU satisfies SU’s data rate requirement. Let $c_{j,t}$ be the number of idle sub-carriers in chunk j detected by the SU sink at time t, and let $C_{j}=\{c_{j,t-g+1},c_{j,t-g+2},\ldots ,c_{j,t}\}$ represent the number of idle sub-carriers in chunk j over the recent g times spectrum-sensing operations. Then, we can determine the parameter for the opportunistic capacity model by applying the point estimation method [32] to achieve the opportunistic capacity of chunk j. Moreover, by adopting the adaptive modulation and coding (AMC) scheme, SUs at different positions can achieve different data rates in the same chunk due to differences in the environment. According to G. J. Foschini’s inference [33], $r_{i,j}$ can be calculated as follows:

(24) $$r_{i,j}=b\log_{2}(1+\frac{\gamma_{i,j}}{-\frac{2}{3}\ln\frac{P_{b}}{2}}),$$

where $P_{b}$ is the maximum tolerable error rate, b denotes the bandwidth of the sub-carrier, and $\gamma_{i,j}=p_{i,j}\cdot H_{i,j}$, which indicates the signal-to-interference plus noise ratio (SINR) when SU i transmits on chunk j. Here, $p_{i,j}$ and $H_{i,j}$ represent the transmit power and the channel factor, respectively.

To explore in which case the proposed allocation model can be used, we analyze the feasibility for it. This analysis also can be used to determine the relationship between the available resources and SUs’ requirements. For the proposed model, the conditions to acquire a feasible solution can be achieved by using expectation constraint method which mainly used in stochastic programming [34]. Due to the randomness of channel condition, the data rate per chunk can achieve different values by adopting the AMC scheme. Thus, we can use random variable to express the data rate of chunks. Based on this, Equations (22) and (23) can be integrated into an expectation constraint which is as follows

(25) $$\sum_{j=1}^{k}c_{j}r_{j,avg}\geq Q,$$

where $r_{j,avg}$ represents the expectation of the data rate for chunk j and $Q=\sum_{i=1}^{m}q_{i}$. Now we can get the relationship between the available resources and SUs’ requirements. This can help us to set model parameters and determine under which condition the feasible solution exists. When the SUs’ requirements are beyond the available resources, Equation (25) cannot be satisfied, and we gradually reduce the access number of SUs to obtain feasible allocation scheme.

### 4.2. Simplification and Solution

The joint power and chunk allocation causes the model to become a mixed integer programming problem, which is hard to solve because the chunk allocation involves 0-1 integer programming and the power allocation involves non-integer programming. One of the direct solution methods is using the heuristic algorithm to realize chunk and power allocation for SUs. The heuristic algorithm exhaustively searches over all possible chunk combinations for SUs and implements power allocation algorithm for each combination. The computational complexity of this algorithm is very high, because the number of combinations is very large and each power allocation is a NP-hard problem. For m SUs and k idle chunks, there exist $m^{k}$ possible combinations. Thus, the heuristic algorithm needs $O(m^{k})$ operations which have NP-hard computational complexity. To reduce the computational complexity, we simplify the model described above by splitting it into two sub-problems. The first step is chunk allocation, which allocates a chunk so a particular user can transmit its data over the chunk. Chunk allocation is based on chunk channel quality, without considering information concerning the transmit power. The second step is power allocation, which allocates the required transmit power over the allocated chunk.

**Figure 3:**
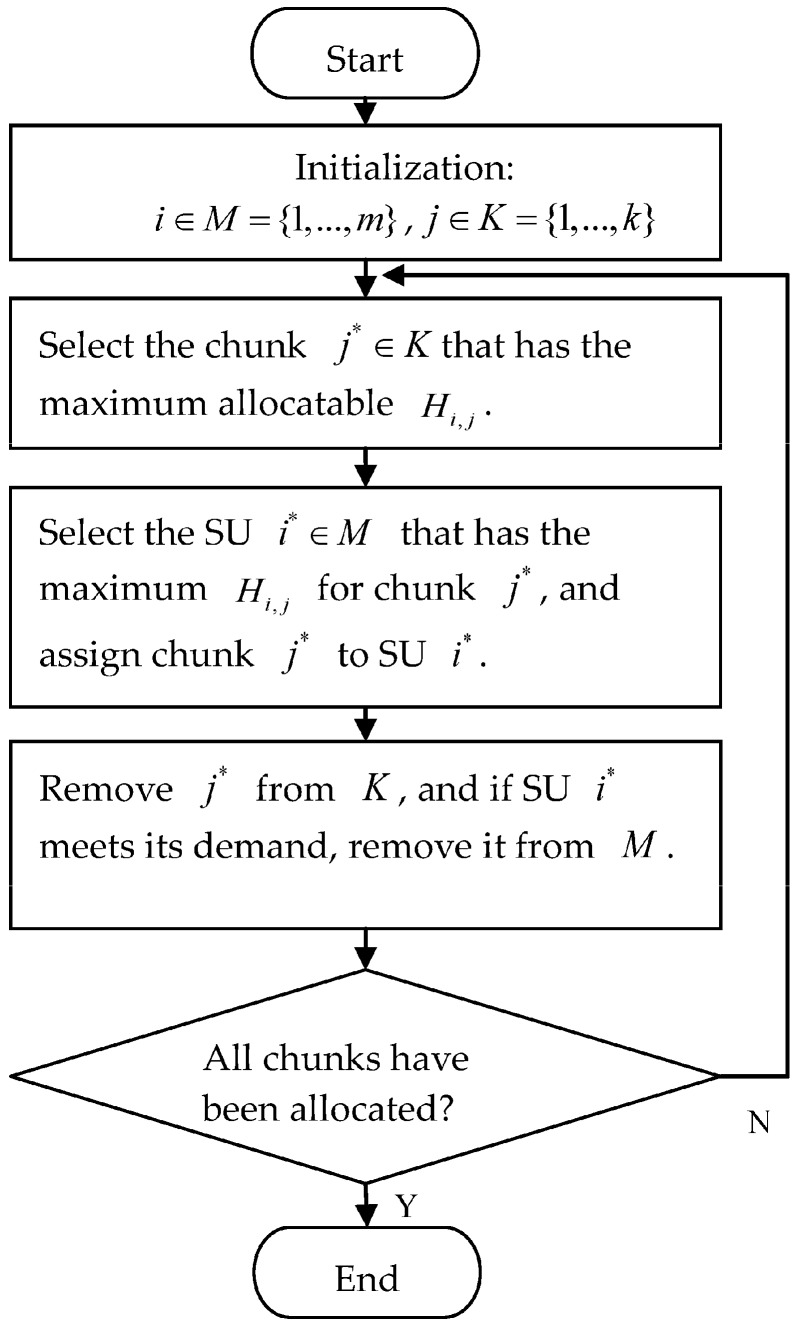
The procedure for chunk allocation.

In the chunk allocation part, we introduce a suboptimal algorithm with low complexity because the integer programming problem with large numbers of variables is difficult to solve. We assume the total transmit power is assigned equally to each chunk with the average power $p_{v}$ and allocate a chunk to a particular SU with the best channel quality to achieve the maximum transmission rate. This procedure is shown in Figure 3. The proposed chunk allocation needs O(kmlog m) operations. After that, the power allocation algorithm only needs to be implemented once. Thus, it has lower computational complexity than the heuristic algorithm.

After all chunks have been allocated, the original objective in Equation (20) leads to power allocation for all chunks based on the previous chunk allocation result. Denoting the chunk allocation result as $X=\{x_{1,1},\ldots ,x_{1,k},\ldots ,x_{m,k}\}$, the optimization problem can be solved by using the Lagrangian dual method [35,36]. For Equation (20), consider the following Lagrangian function:

(26) $$L(P,\lambda ,\mu )=\sum_{i=1}^{m}\sum_{j=1}^{k}c_{j}r_{i,j}x_{i,j}+\lambda (P_{T}-\sum_{i=1}^{m}\sum_{j=1}^{k}p_{i,j}x_{i,j})+\sum_{i=1}^{m}\mu_{i}(\sum_{j=1}^{k}c_{j}r_{i,j}x_{i,j}-q_{i}),$$

where $P={\left[p_{1,1},\ldots ,p_{m,k}\right]}^{T}\geq 0$. λ≥0 and $\mu ={\left[\mu_{1},\ldots ,\mu_{m}\right]}^{T}\geq 0$ are the Lagrange multipliers for Constraints (21) and (22), respectively. The Lagrangian dual objective function is

(27) $$\theta (\lambda ,\mu )=\underset{\lambda ,\mu \geq 0}{\max}\;L(P,\lambda ,\mu ).$$

Hence, the Lagrange dual problem can be obtained as follows

(28) $$\min\theta (\lambda ,\mu )=\min\underset{\lambda ,\mu \geq 0}{\max}\;L(P,\lambda ,\mu ).$$

The derivative of L(P,λ,μ) is

(29) $$\frac{\partial L}{\partial p_{i,j}}=c_{j}x_{i,j}\frac{dr_{i,j}}{dp_{i,j}}-\lambda x_{i,j}+\mu_{i}c_{j}x_{i,j}\frac{dr_{i,j}}{dp_{i,j}},$$

where $\frac{dr_{i,j}}{dp_{i,j}}$ can be obtained by differentiating Equation (24) with $p_{i,j}$.

(30) $$\frac{dr_{i,j}}{dp_{i,j}}=\frac{bH_{i,j}}{(-\frac{2}{3}\ln\frac{P_{b}}{2}+p_{i,j}H_{i,j})\ln2}.$$

Substituting Equation (30) into Equation (29) yields

(31) $$\frac{\partial L}{\partial p_{i,j}}=\frac{c_{j}bH_{i,j}x_{i,j}}{(-\frac{2}{3}\ln\frac{P_{b}}{2}+p_{i,j}H_{i,j})\ln2}-\lambda x_{i,j}+\frac{\mu_{i}c_{j}bH_{i,j}x_{i,j}}{(-\frac{2}{3}\ln\frac{P_{b}}{2}+p_{i,j}H_{i,j})\ln2}.$$

Thus, the solution for the optimal power allocation based on the chunk allocation result can be derived by setting $\frac{\partial L}{\partial p_{i,j}}=0$. Then, the transmit power of chunk j for SU i is

(32) $$p_{i,j}=\frac{C_{j}bH_{i,j}(1+\mu_{i})+\frac{2}{3}\lambda \ln2\ln\frac{P_{b}}{2}}{\lambda H_{i,j}\ln2}.$$

Taking Equation (32) into Equation (28), the result is

(33) $$\min\theta (\lambda ,\mu )=\underset{\lambda ,\mu \geq 0}{\min}L(\lambda ,\mu ).$$

Subsequently, the problem can be solved by using gradient method. According to the gradient method and Equation (32), the iterative formulas for μ and λ are

(34) $$\lambda (t+1)={\left[\lambda (t)-\alpha (t)(P_{T}-\sum_{i=1}^{m}\sum_{j=1}^{k}p_{i,j}x_{i,j})\right]}^{+},$$

and

(35) $$\mu_{i}(t+1)={\left[\mu_{i}(t)-\beta_{i}(t)(\sum_{j=1}^{k}c_{j}r_{i,j}x_{i,j}-q_{i})\right]}^{+},$$

respectively, where ${\left[\cdot \right]}^{+}=\max(0,\cdot )$, t is the iteration number, and α(t) and $\beta_{i}(t)$ denote the step sizes for each iteration. Based on this, by selecting a sufficiently small step size, the gradient method convergence is guaranteed and the optimal power allocation will be achieved [37]. The pseudocode for solving algorithm shown in Algorithm 1.

**Algorithm 1:** The pseudocode for solving algorithm: two step solving algorithm
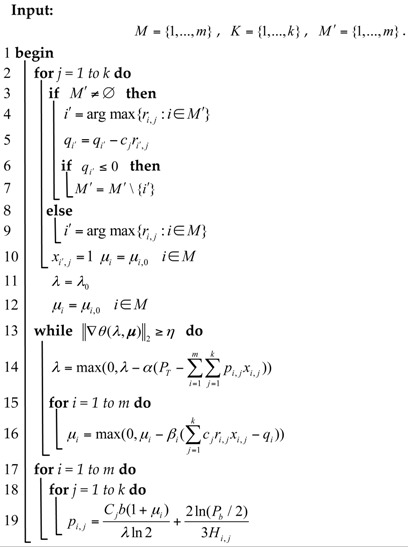


## 5. Simulation Results

This section investigates the performance of the proposed opportunistic capacity-based resource allocation algorithm using numerical simulations. We assume that all SUs are randomly located around the SU sink and that the simulation parameters are set as shown in Table 1. To simulate the time-varied spectrum environment caused by PU/SU activity and mobility, we randomly generate spectrum holes and divide them into chunks in which the available capacity changes based on a chunk capacity change rate p (which denotes the probability of the chunk capacity changing in the next moment). Then, we compare the performance of the proposed opportunistic capacity-based resource allocation algorithm with the traditional chunk-based resource allocation [24] and the sub-carrier-based resource allocation algorithm (n=1).

**Figure 4:**
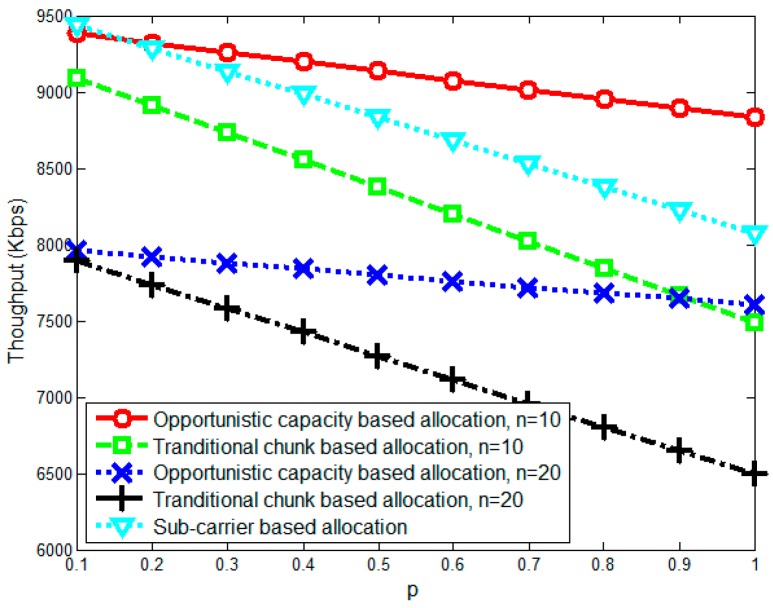
Average throughput with different chunk capacity change rates p.

Figure 4 depicts the average throughput with different chunk capacity change rates p for different chunk sizes (n=10 and n=20). In Figure 4, as p increases, the average throughput decreases noticeably due to bandwidth collisions caused by the fast spectrum changes. The sub-carrier-based allocation has similar behavior as the opportunistic capacity-based allocation (n=10) in p<0.3 part because of the flexible sub-carrier allocation. However, as p increases, the sub-carrier-based allocation has a serious performance degradation because it only considers current static optimization and can hardly adapt the dynamic spectrum environment. Moreover, compared with the chunk-based allocation, the sub-carrier-based allocation introduces more computational complexity since the assignable units increased by n times. The traditional chunk-based allocation method achieves lower average throughput than does the opportunistic capacity-based allocation method because the former considers only the current static optimal, and it uses constant allocation parameters, which leads to more bandwidth collisions. In the n=10 scenario, the throughput gap between the opportunistic capacity-based allocation and traditional chunk-based allocation is small when p=0.1. However, as p increases, the opportunistic capacity-based allocation method increasingly outperforms the traditional chunk-based allocation, which shows that the opportunistic capacity-based allocation method adapts better to situations with frequency time-varied spectrum environments and can achieve high spectrum efficiency. This occurs because the traditional chunk-based allocation method is unable to follow the fast-changing spectrum environment in real time. The spectrum changes occur quickly, but the opportunistic capacity-based allocation, using its dynamic optimization method, reduces bandwidth collisions by considering the statistical properties of chunks. In addition, when the number of sub-carriers in chunks increase (the n=20 scenario), the average throughput is lower than when n=10, showing that large chunks reduce the throughput because they are less flexible. Besides, because there are massive rounds of spectrum changes (1000 rounds in our simulation), the average throughput will approach the values achieved by taking the expectation of chunk capacity as the changed chunk capacity. So the average throughput approaches a linear manner since p changed in a linear manner.

**Figure 5:**
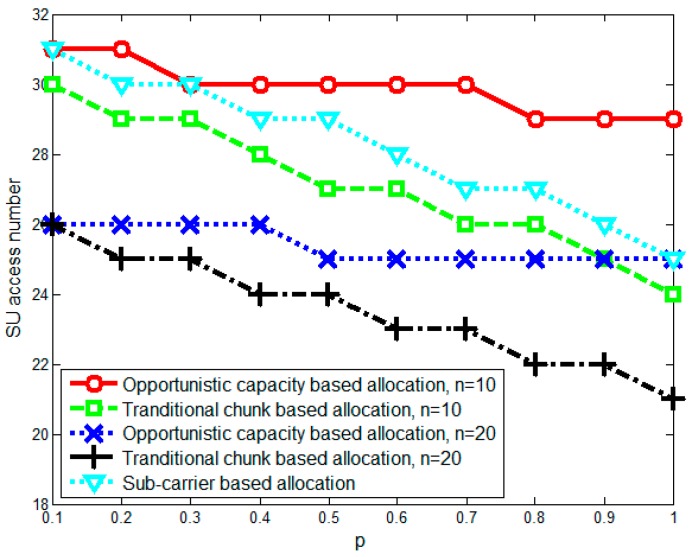
Number of secondary users (SUs) accessing the network with different chunk capacity change rates p.

Figure 5 shows the number of SUs accessing the network at different chunk capacity change rates p and different chunk sizes (n=10 and n=20). In the figure, as p increases, the SU access number decreases due to bandwidth collisions. The sub-carrier-based allocation achieves well performance in p<0.3 part due to the flexible sub-carrier allocation, but its performance decreased rapidly as p increased. This is because the spectrum changes will cause the sub-carrier collisions and greatly degrade its performance. The opportunistic capacity-based allocation scheme behaves well in this scenario, which has a time-varied spectrum environment. This occurs because compared with the traditional chunk-based allocation and the sub-carrier-based allocation methods, opportunistic capacity-based allocation takes the opportunistic capacity of chunks into consideration and, thus, can ensure that the SUs’ data rate requirements are met and also reduce the impact of bandwidth collisions when the spectrum environment changes. In addition, the number of SUs accessing the network under the opportunistic capacity-based allocation method falls less as p increases in both the n=10 and n=20 scenarios. This result shows that the proposed method can obtain good performance with different chunk sizes.

**Figure 6:**
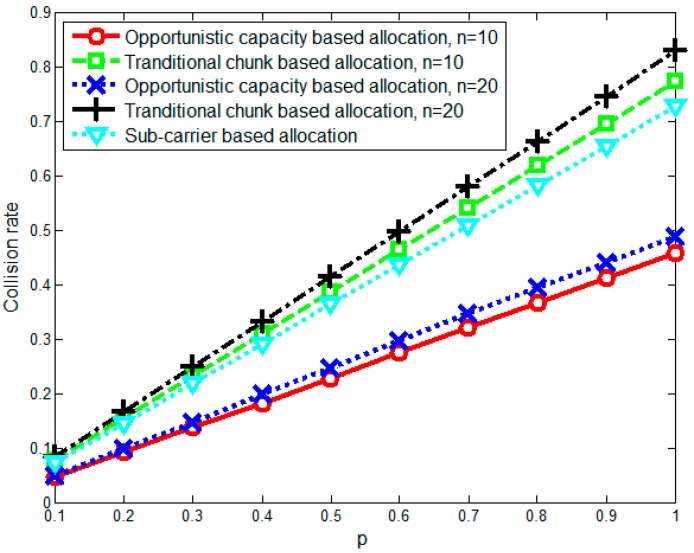
Average spectrum collision rate with different chunk capacity change rates p.

Figure 6 shows the average spectrum collision rate with different chunk capacity change rates p for different chunk sizes (n=10 and n=20). From Figure 6, we can see that as p increases, the spectrum collision rate increases dramatically due to the fast-changing spectrum environment. In such scenarios, the opportunistic capacity-based allocation method achieves a lower collision rate than the traditional chunk-based allocation and sub-carrier-based allocation. This occurs because the opportunistic capacity-based allocation reduces bandwidth collisions by assigning spectrum resources while considering the statistical properties of chunks. This result shows that opportunistic capacity-based allocation can effectively minimize the spectrum collision rate when the spectrum environment changes. In the n=20 scenario, the spectrum collision rate of the opportunistic capacity-based allocation almost the same as when n=10, which also implies that the spectrum collision rate is not susceptible to variations in chunk size. In addition, the average spectrum collision rate approaches a linear manner because of the similar reasons for the average throughput.

## 6. Conclusions

In this paper, we investigated resource allocation for chunk-based multi-carrier CRSNs, where the available spectrum resources vary over time due to PU/SU activity and mobility. We presented a novel opportunistic capacity model using a CTSMC to describe the time-varied spectrum resources of chunks. This approach established a joint power and chunk allocation model based on the opportunistic capacity of chunks for chunk-based multi-carrier CRSNs with time-varied spectrum resources. To reduce the computational complexity, we split this model into two sub-problems and solved them via the Lagrangian dual method. Simulation results showed that the proposed opportunistic capacity-based resource allocation achieves better performance than the traditional chunk-based allocation algorithms in time-varied spectrum environments.

## Figures and Tables

**Table 1:** Simulation parameters.

Parameters	Values
Maximum tolerable error rate $P_{b}$	$10^{-6}$
SU number AMUN	40
Sub-carrier bandwidth BW	20 KHz
Sub-carrier number S	400
Sub-carrier number in each chunk n	10–20
Rician fading channel factor K	3
Noise power $N_{0}B$	−111 dBm
Chunk capacity change rate p	0.1~1
